# Systematic Review of En Bloc Resection in the Management of Ewing's Sarcoma of the Mobile Spine with Respect to Local Control and Disease-Free Survival

**DOI:** 10.1097/MD.0000000000001019

**Published:** 2015-07-13

**Authors:** Mathew David Sewell, Kimberly-Anne Tan, Nasir A. Quraishi, Corina Preda, Peter P. Varga, Richard Williams

**Affiliations:** From the Princess Alexandra Hospital, Brisbane, Australia (MDS, CP, RW); University of New South Wales, Faculty of Medicine, Sydney, Australia (K-AT); Centre for Spine Studies and Surgery, Queens Medical Centre, Nottingham, UK (NAQ); and The National Centre for Spinal Disorders, Budapest, Hungary (PPV).

## Abstract

There is no consensus on the optimal method of local control in Ewing's sarcoma (ES) of the mobile spine. Recent reports have suggested that en bloc resection may improve local control and survival. The authors therefore performed a systematic review to answer the following questions: (1) What is the outcome of en bloc resection for ES of the mobile spine with respect to local control and disease-free survival (DFS)? (2) How should residual ES of the mobile spine be treated?

Inclusion criteria were articles published between the years 1960 and 2014 in English that contained more than five patients. This yielded 204 articles, from which 4 were selected for detailed analysis. The literature was graded for quality, summarized, and presented to a group of spinal oncology experts with consensus recommendations made.

All 4 studies were retrospective case series graded as very low quality evidence. Local control strategies included radiotherapy (RT) alone, surgery and RT, or surgery alone. There was no standardized outcome reported across studies with respect to the type of surgical procedure, margins, and outcomes of interest such as local recurrence (LR) and DFS. When the en bloc procedures were pooled together, 2 of the 21 patients with available LR data developed LR (9.5%), and 5 of the 7 patients with available DFS data were disease free at a mean of 76 months. The remaining 2 died at 10 and 29 months, respectively. No studies were identified detailing the treatment of residual ES of the mobile spine.

There is no consensus on the optimal method of local control for spinal ES or the treatment of residual disease. A weak recommendation supports that when the en bloc resection is technically possible, in combination with RT, this appears to provide superior local control than RT alone, or incomplete excision and RT. The effect on survival is indeterminate.

## INTRODUCTION

First described by James Ewing in 1921, Ewing's sarcoma (ES) is the second most common primary malignant bone cancer in children and adolescents, after osteosarcoma.^1^ Histologically ES comprises small round blue cells originating from bone and soft tissues; the expression of neural markers distinguishes conventional ES from malignant primitive neuroectodermal tumors (PNET). The most common primary sites of involvement are the pelvis, femur, and tibia. Primary spinal involvement is rare with a reported incidence of 5% in the mobile spine (cervical, thoracic, and lumbar).^2^

The introduction of modern, multiagent chemotherapy, combined with radiotherapy (RT) and/or surgery for definitive local control, has appreciably improved disease-free survival (DFS) to 50–80%.^3^ However, in the limited number of retrospective reviews published, the prognosis is still considered worse in the spine, and there is no consensus on the optimal method of local control.^4^ Radiotherapy is currently the main component of local therapy, but its use is restricted in dose and extent, owing to proximity to the spinal cord, and in lumbar tumor sites, the kidneys.

The majority of published reports detailing outcome of surgery for local control in the spine do not apply the same surgical oncological principles as those utilized in the limb. In the long bones, surgical resection obeying Enneking's principle of “en bloc” resection, aiming to remove the tumor as a whole, fully covered by a continuous shell of healthy tissue, has improved survival, in addition to the local control rate.^5^ In the spine, wide en bloc resection is not always possible because of proximity to local neural structures, and possibly lack of surgical experience with “en bloc” vertebral resection techniques as described by Tomita.^6^ Historically, the majority of surgical procedures performed for spinal ES have been intralesional (IL) procedures (surgical debulking or decompressive laminectomy). These IL procedures do not fulfill the Enneking principle of surgical resection and may have negatively biased the results of surgery as a method of local control, compared to RT.

There is very little in the literature on the outcome of surgical treatment of spinal ES, and, even less on the outcome of en bloc resection. In view of recent literature reports postulating that en bloc resection may improve local control,^4^ we therefore performed a systematic review to answer the following two questions:

1. What is the outcome of en bloc resection for ES of the mobile spine with respect to local control and DFS?
2. How should residual ES of the mobile spine be treated?

## METHODS

An electronic literature review was conducted via PubMed, Ovid Medline, Web of Science, EMBASE, and the Cochrane Library using the search terms “Ewing's sarcoma” or “Ewing sarcoma” AND “spine,” and “local recurrence,” or “radiotherapy,” or “survival,” or “surgery,” “treatment,” or “en bloc resection.” These searches yielded 204 articles. All searches were conducted in July 2014 and the search was limited to English language articles. Results were screened based on their abstracts, in a non-blinded fashion by 2 reviewers. The remaining papers were reviewed to determine suitability for inclusion. Following review of the reference lists of these articles, an additional paper was included. Ethical approval was not required for this literature review.

Inclusion criteria were articles published between 1960 and 2014 in English that contained >5 patients and also contained information on the type of surgical resection for local control (IL, marginal, en bloc with wide margins) and outcome (local recurrence and DFS) in the mobile spine. Publications with insufficient information on the type of surgical resection and associated outcome were excluded. “En bloc resection” was defined as tumor excision covered in whole by a continuous shell of healthy tissue. Decompressive laminectomy (DL) and debulking were considered IL surgical procedures, whereas open biopsy was not. Primary outcome measures examined were local recurrence (LR) and DFS. No randomized control trials were identified, and despite liberal inclusion criteria, retrospective case series were the main article types identified for inclusion. As standardized outcome reporting across studies was lacking, we pooled together en bloc procedures and procedures with wide or marginal resection margins that had specific individualized data on LR and/or DFS.

Publications were classified as the level of evidence high, moderate, low or very low, based on GRADE determinants as described by Schunemann et al (2006).^7^ Recommendations were then made by using a modified Delphi technique^8,9^ incorporating the GRADE approach, which weighs quality of evidence with balance of benefits and downsides (harms, burden, and cost). Recommendations may be classified strong or weak.

## RESULTS

1. What is the outcome of en bloc resection for ES of the mobile spine with respect to local control and DFS?
2. How should residual ES of the mobile spine be treated?

Four studies addressing the first question were identified (Table 1  ). All studies were retrospective case series classified as low-level evidence. No studies addressing the second question could be identified.

**TABLE 1:**
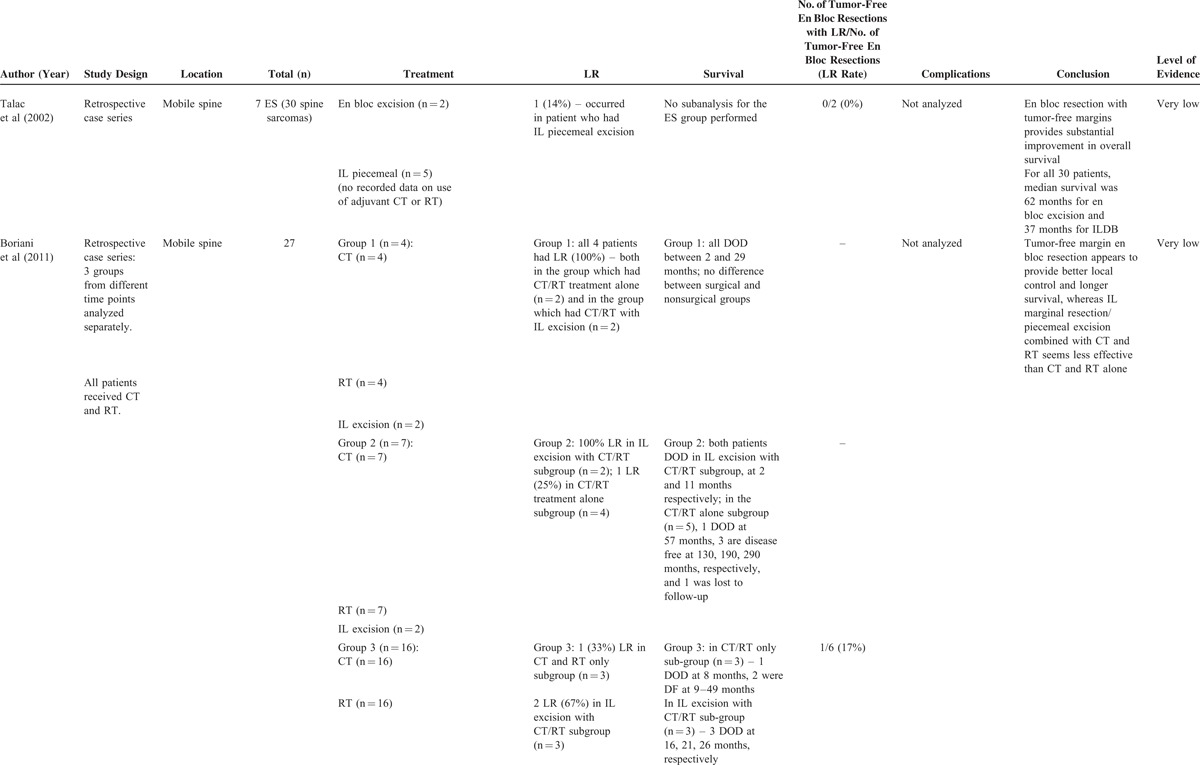
Included Studies in Review

**TABLE 1 (Continued):**
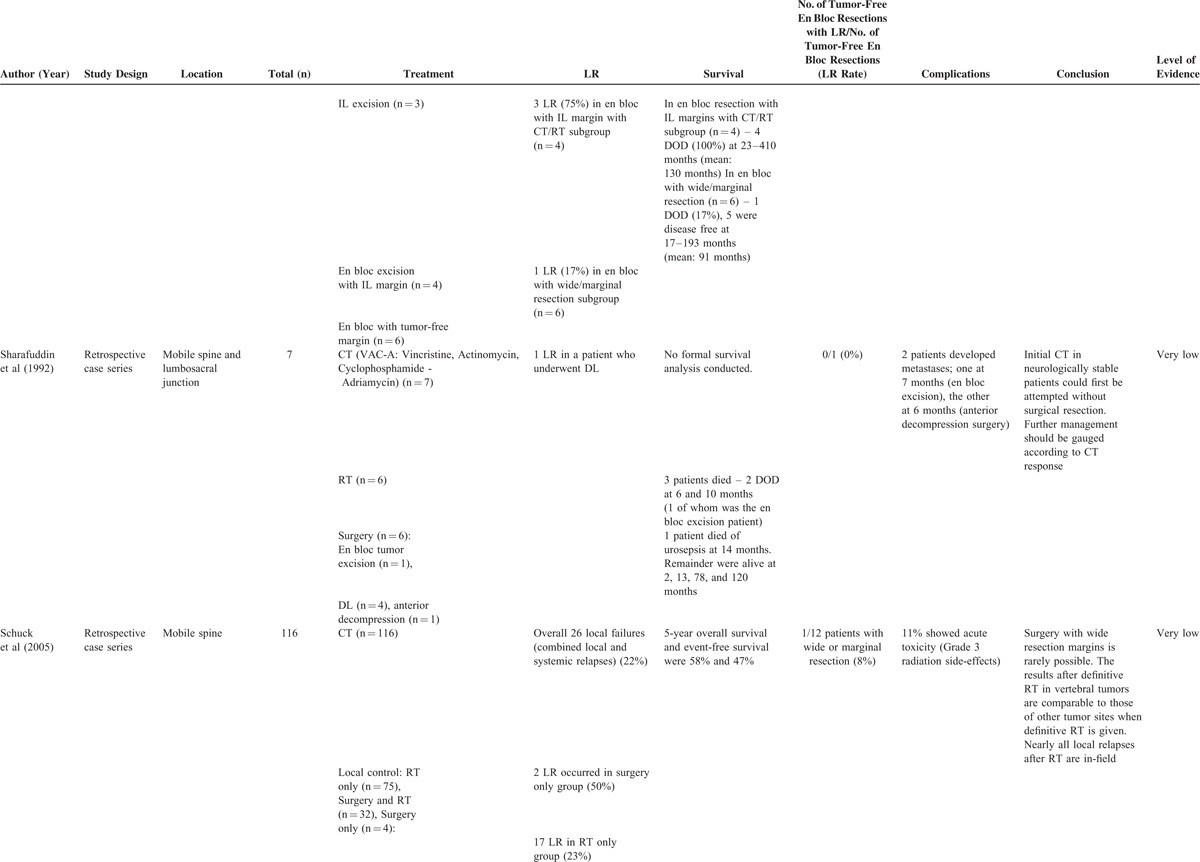
Included Studies in Review

**TABLE 1 (Continued):**
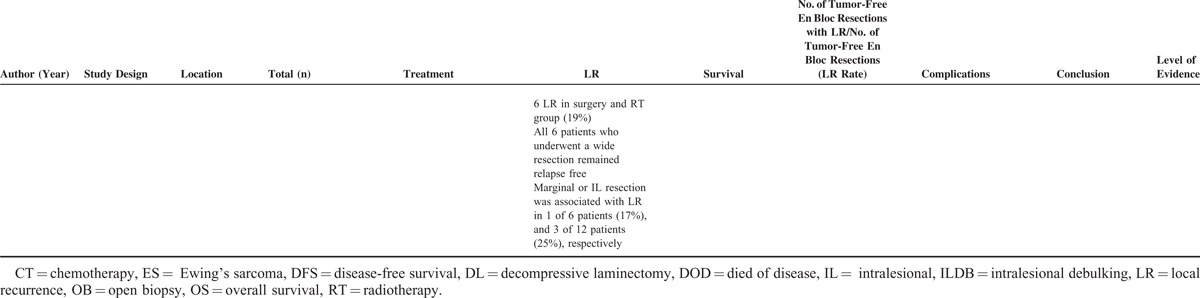
Included Studies in Review

Talac et al (2002)^10^ reviewed 30 primary spine sarcomas, 7 of which were ES. Treatment consisted of en bloc resection in 12 patients (40%) and piecemeal resection in 18 (60%). There was an increased risk of LR in cases with positive margins versus cases with negative margins. LR resulted in disease progression and death in 11 of 12 patients (92%). Median survival was 62 months for those who had en bloc resection, and 37 months for those with piecemeal resection. No information was provided regarding adjuvant treatments (CT/RT), and the high proportion of chondrosarcomas (27%) may have skewed overall analysis, which did not stratify tumors by subtype. Subtype analysis showed that of the 5 ES patients who underwent piecemeal excision, 1 developed LR (20%). Neither of the 2 who had en bloc resections developed LR. DFS for the subgroup of ES patients was unreported.

Boriani et al (2011)^11^ retrospectively reviewed 27 patients over three separate time periods to evaluate the role of en bloc resection on the oncological outcome of patients with ES treated with systemic CT combined with RT. In the first period between 1979 and 1982, 4 patients were treated with CT (REA-2) and RT. Two underwent IL excision. All patients died 2–29 months later with no difference between surgical and nonsurgical groups. Between 1983 and 1990, 7 patients were treated with CT (REN-1/2) and RT. Two underwent IL excision, had worse disease evolution, and died of disease (DOD) at 2 and 11 months. The patients who did not undergo surgery evolved more favorably: 1 DOD at 57 months, and 3 were disease free at 130, 190, and 290 months.

In the final period between 1991and 2008, 16 patients were treated with CT (REN-3/ISG-SSG) and RT, combined with IL excision in 3, en bloc with IL margins in 4 and en bloc with tumor-free margins (marginal or wide) in 6; LR rates were 33% for the CT and RT only groups, and 67%, 75% and 17% for the remaining groups respectively. One patient submitted to tumor-free margin en bloc resection developed LR and DOD at 29 months. This patient had previously undergone an open biopsy. The remaining patients who underwent tumor-free margin en bloc resection were disease free at 17, 22, 37, 188, and 193 months (mean: 76 months). All the patients submitted to IL excision and to en bloc resection with margin violation had similar prognosis, as they DOD 10–63 months after treatment. Only 1 of 3 patients who had no surgery DOD 8 months after treatment, the others surviving 9 and 49 months follow-up. This study suggested tumor-free margin en bloc resection provided better local control and longer survival, whereas the results of IL resection were worse than CT and RT alone.

Sharafuddin et al (1992)^12^ reported on 7 patients treated with systemic CT (VAC-A) in combination with RT in 6 and surgery in 6 (4 laminectomies with tumor excision, 1 anterior decompression with tumor excision, and 1 en bloc tumor excision). LR occurred in a patient who had undergone laminectomy and adjuvant RT. Three patients died: 2 DOD at 6 and 10 months (one of these was the patient who underwent en bloc excision), and a 3rd patient died of urosepsis. The DFS in the patient who underwent en bloc resection was 7 months.

Schuck et al (2005)^13^ reviewed 116 patients with ES of the mobile spine who all underwent CT as part of the cooperative Ewing's sarcoma study. For local treatment, 75 had RT alone, 32 RT and surgery and 4 surgery alone. There were 26 failures of local therapy (22%); 2 of these occurred in the surgery only group (50%), 17 in the RT only group (23%), and 6 in the surgery and RT group (19%). One LR occurred in a patient with unspecified local therapy. Differences in LR between these treatment groups were not significant. There was no specific information provided on the type of surgeries performed. However, information was provided on associations between margins and LR: all 6 patients who had a wide resection remained relapse free, marginal resection was associated with LR in 1 of 6 patients (17%), and IL resection with LR in 3 of 12 patients (25%). Five-year overall survival and DFS were 58% and 47%, respectively. No information on DFS for the subgroup of wide resection patients was available. The authors concluded that surgery with wide resection margins is rarely possible. The results after definitive RT in vertebral tumors are comparable to those of other tumor sites treated with definitive RT. Nearly all local relapses after RT are in-field.

Although there was no standardized outcome reporting across studies with respect to the type of surgical procedure, margins, and outcomes of interest (LR/DFS), we pooled together the en bloc procedures, and procedures with wide resection margins, that had specific individualized data on LR. This yielded 21 patients, of whom 2 (9.5%) developed LR. We also pooled together the en bloc procedures, and procedures with wide resection margins that had specific individualized data on DFS. This yielded 7 patients, 2 of whom had died at 10 and 29 months. The remaining 5 had a mean DFS of 76 months.

Following analysis of the literature using the GRADE approach, consensus recommendations incorporating a modified Delphi technique were made.

Recommendation 1: there is no consensus on the optimal method of local control for spinal ES. When en bloc resection is technically possible, in combination with RT, this appears to provide superior local control to RT alone, or incomplete excision and RT (weak recommendation, very low quality evidence). The effect on survival is indeterminate because of the lack of evidence.

Recommendation 2: there is no information to advise on management of residual ES of the spine. Chemotherapy offers significant improvements in local control and survival and should be the mainstay of treatment. En bloc surgery with wide margins in combination with RT is likely to offer the best chance of definitive local control. When en bloc surgery is not possible, RT alone should be administered with surgery reserved for those requiring decompression of neurological structures.

## DISCUSSION

This systematic review was designed to answer the questions: (1) What is the outcome of en bloc resection for ES of the mobile spine with respect to local control and DFS? And (2) how should residual ES of the mobile spine be treated? With respect to the first question, the level of evidence was very low. Of the en bloc procedures analyzed, 2 of the 21 patients with available LR data developed LR (9.5%), and 5 of the 7 patients with available DFS data were disease free at a mean of 76 months. Therefore, although en bloc tumor resection does not necessarily eliminate LR, it may provide a more effective method of local control compared with RT alone or combined RT and intralesional surgical procedures. We were unable to find any literature regarding the second question on the management of residual ES.

Ewing's sarcoma is rare. In the United States, ∼200 cases are diagnosed annually,^14^ of which <30 involve the mobile spine,^15^ as only ∼5% of ES affects the mobile spine.^2^ Continual advancement of systemic therapy, such as the recent discovery of PARP inhibitor efficacy,^16^ will maintain CT as the most significant factor for improving survival.^3^ Additionally, patients who undergo surgery are also thought to demonstrate improved survival.^17^ However, the optimal method of local control in spinal disease remains to be indeterminate. This is in part because of methodological challenges related to low patient numbers, selection bias, lack of standardized outcome reporting across studies, and no standardized treatment methods for local control.

Unlike peripherally located disease, local control methods for spinal disease are frequently dictated by neurological symptoms at presentation before classical decisional factors such as tumor location, size, and response to CT. In the presence of acute neurological deficit secondary to epidural compression, IL decompressive procedures with biopsy are most commonly performed with RT as an adjunct. This may relieve neurological symptoms in >50%,^15^ but deviation from Enneking principles predisposes to LR and metastasis.

In neurologically stable patients, local control options include surgery alone, RT alone, or a combination of both. Radiotherapy is currently considered the backbone of successful local treatment;^18^ however, adequate treatment can require up to 50–60 Gy, exceeding dose tolerance of the spinal cord (55 Gy).^19^ The role of surgery remains unresolved, as some studies report that IL marginal resection, or piecemeal excision combined with CT and RT may be less effective than CT and RT alone.^11^

A systematic review by Sciubba et al (2009),^4^ involving patients with ES of the axial and appendicular skeleton, suggested that more aggressive surgical resection was associated with improved overall survival and local control. It is important to note that these conclusions were mainly based upon evidence for tumor resections involving the limbs, rather than the spine. Based on very low quality evidence, a team of experts from the AO spine tumor oncology group postulated that en bloc resection may provide improved local control for ES of the spine, but not improved overall survival. They also recommended that RT may be used for local control either alone or to supplement incomplete resection.

The overall 5-year relative survival rates for spinal ES varies between 30% and 65%.^20–23^ Interestingly, the mean DFS in 5 of the 7 patients who underwent en bloc resection in this review was 76 months, which is favorable in comparison to other studies, although this may reflect selection bias.

Comparing studies in which patients were treated with CT plus RT alone for definitive local control of spinal ES, Marco et al (2005)^21^ reported a 23% LR and 36% 10-year DFS in 13 patients. Venkateswaran et al (2001)^24^ reported similar rates in 33 patients treated with RT alone for definitive local control. Most studies report outcomes on patients treated with a combination of RT and surgery for definitive local control. Vogin et al (2013)^18^ reported LR in 19 of 75 patients (25%) at a median time of 25 months in patients treated with RT alone (19), a combination of surgery and RT (50) or surgery alone (6), with 66% of the surgeries being IL. Paulino et al (2007)^20^ reported a higher LR rate (36%) in 11 patients treated by similar methods. Indelicato et al (2010)^15^ reported a 10% LR rate and 47% 5-year DFS in 27 patients treated by either RT alone or surgery and RT. Local control was superior in those treated with surgery and RT.

The comparison of results between studies in the literature is not accurate because of varying treatment protocols, selection bias, and lack of standardized outcome reporting. Prognosis of ES is significantly influenced by the use of CT.^16,25–30^ Included papers in this review reported differing CT regimens, reflecting changes in CT use in ES. There are many confounding factors such as age, timing of surgery, neurological status, presence of metastases, and response to CT that could not be factored into the analysis. This, in addition to the small number of included papers, limits the interpretation of results in this review. Furthermore, en bloc resection is only applicable to a small subset of patients with spinal ES, as resection obeying Enneking's principles is not always possible because of technical difficulties in obtaining wide margins around the spinal canal.

## CONCLUSION

Based on very low quality evidence, the expert panel opinion in this review supports en bloc resection (when technically achievable) in combination with RT, as this appears to provide superior local control to RT alone, or incomplete excision and RT, with an indeterminate effect on survival. It should be remembered that morbidity and mortality of en bloc resection is significant^31^ and should only be performed by multidisciplinary teams. As improvements in systemic therapy continue to extend the survival of children and adolescents with ES, there is imperative for more uniform endpoint reporting of different methods of local control, in order to further improve outcomes.
